# Acquiring a Pet Dog Significantly Reduces Stress of Primary Carers for Children with Autism Spectrum Disorder: A Prospective Case Control Study

**DOI:** 10.1007/s10803-015-2418-5

**Published:** 2015-04-02

**Authors:** H. F. Wright, S. Hall, A. Hames, J. Hardiman, R. Mills, PAWS Team, D. S. Mills

**Affiliations:** 1 School of Life Sciences, Joseph Banks Laboratories, University of Lincoln, Lincoln, LN6 7DL UK; 2 Research Autism, Adam House, 1 Fitzroy Square, London, W1T 5HE UK; 3 Department of Psychology, University of Bath, Bath, UK; 4 Dogs for the Disabled, Frances Hay Centre, Banbury, Oxfordshire, OX17 2BS UK

**Keywords:** ASD, Autism, Child, Family, Carer, Dogs, Intervention

## Abstract

This study describes the impact of pet dogs on stress of primary carers of children with Autism Spectrum Disorder (ASD). Stress levels of 38 primary carers acquiring a dog and 24 controls not acquiring a dog were sampled at: Pre-intervention (17 weeks before acquiring a dog), post-intervention (3–10 weeks after acquisition) and follow-up (25–40 weeks after acquisition), using the Parenting Stress Index. Analysis revealed significant improvements in the intervention compared to the control group for Total Stress, Parental Distress and Difficult Child. A significant number of parents in the intervention group moved from clinically high to normal levels of Parental Distress. The results highlight the potential of pet dogs to reduce stress in primary carers of children with an ASD.

## Introduction

Autism spectrum disorder (ASD) is a heterogeneous condition defined by the DSM-5 as a person experiencing persistent difficulties in social interaction in a range of contexts and as showing restricted, repetitive behaviours. These problems must have been evident in early childhood, cause significant impairment in functioning and not be explainable by intellectual disorders or developmental delays (DSM-5, APA 2013). Parenting children with developmental disorders, such as ASD is associated with higher levels of stress, anxiety and negative outcomes (such as depression and social isolation) when compared to parenting typically developing children, or children with other non-developmental disabilities (Dunn et al. 2001; Koegel et al. 1992; Weiss et al. 2013; Wolff et al. 1989). High levels of stress impact not only on the health and wellbeing of the carers themselves, but can also limit the effectiveness of the outcomes of ASD interventions (Robbins et al. 1991; Osborne et al. 2008). As such the assessment of interventions and lifestyle choices that effectively reduce carer stress is a critically important area for research in ASD treatment programmes.

The availability of social support and levels of stress experienced have been associated with successful adaptation of the carer (Koegel et al. 1992; Konstantareas and Homatidis 1989; Weiss et al. 2013). It has been suggested that the type of social support may relate to the effectiveness of stress buffering, with more informal social support (e.g. spouse, family, friends) acting as a more effective stress buffer compared to formal or structured social support (e.g. parenting support groups) (Boyd 2002). Given that informal social support may be an effective remediation tool for reducing stress in carers it is appealing to investigate the potential of companion animals, such as pet dogs to provide informal social support for parents of children with ASD.

It could be proposed that because carers already experience high levels of physical and emotional demands, the demands of acquiring a pet might increase rather than reduce stress. However, there is increasing evidence to support stress reducing and health enhancing benefits of pets on individuals and families (Allen et al. 2001; Friedman and Thomas 1995). Dog ownership has been found to be a positive factor in supporting individuals in difficult times, including children affected by serious illness and death of a parent (Raveis et al. 1993) and in reducing the symptoms of physical and psychological illness in women coping with the loss of a loved one (Barker and Barker 1988). Furthermore, companion animals have been shown to reduce the onset and severity of stress-related conditions (Wilson 1991). Additionally, there is evidence to support the utility of trained assistance dogs as therapy for children with ASD (Solomon 2010; Berry et al. 2013), with benefits such as enhancing family freedom (Burrows et al. 2008), reducing child stress (Viau et al. 2010) and improving the effectiveness of therapy sessions (Silva et al. 2011). These effects are remarkable in themselves for the benefits they provide for the child with ASD, but they may also induce a wider positive impact upon parental stress levels, particularly in circumstances where the dog lives as part of the family as opposed to being a part of structured therapy sessions. Indeed, a study by Burgoyne et al. (2014) report an increase in caregiver competence, although not caregiver strain, in families living with a trained assistance dog.

The limited number of studies which have explored the effects of pet dogs in families with children with ASD, have primarily focused on outcomes related to the child with the ASD diagnosis. For instance, a recent study by Grandgeorge et al. (2012) showed that parents reported an increase in prosocial behaviours in their child with ASD and a reduction in anxiety with the acquisition of a pet (including cat, dog and small furry animal). Such beneficial effects in child behaviours may improve parental stress. Alternatively, because pet dogs interact with the entire family unit it is also possible that the dog may elicit similar stress reducing effects directly in the parent. The only other (known) study looking at the effects of pet (as opposed to service/assistance) dogs reports parental opinions on their perceived benefits and limitations of acquiring a dog in the family (Carlisle 2014). Indeed, it is important that parents acquire a dog after careful consideration of both the potential benefits and negative implications of dog ownership. Due to both the individual nature of ASD and the characteristics of dogs as unique living species it is unlikely that dog ownership will benefit all families in the same way. Therefore, whilst the purpose of this paper is to report data illustrating positive effects of pet dogs for parents of children with ASD we are keen to point out pet dogs as effective ASD therapy is still in its infancy and requires greater scientifically robust evaluations. Our research team has investigated parental expectations of acquiring a pet dog and observed some noticeable disparities between expectations and reality which are important for practitioners and parents to consider when thinking about dog ownership (Wright, Hall, Hames, Hardiman, Burgess, Mills and Mills, under review).

As previously mentioned parental stress levels are thought to be important determiners in the success of ASD therapy programmes (Robbins et al. 1991; Osborne et al. 2008), therefore, one important stage in developing understanding of animal companionship in ASD therapy is to investigate the impact of dog ownership on parent stress. Given the potential combined benefits for the parent, child and whole family, pet dogs may be a widely acceptable and flexible lifestyle change which reduces stress in the carers of children with ASD. Furthermore, there are many potential benefits of dogs as an intervention in this context; they are accessible, socially valid and acceptable in most western cultures, therefore, the aim of this study was to assess the impact of acquiring a family pet dog on the stress of carers of a child with ASD.

## Methods

The research process was approved by the University of Lincoln’s ethics committee.

### Participants

Participants were recruited to take part in the study if their child had a confirmed diagnosis of autism spectrum disorder. Because of the heterogeneous nature of ASD we did not include a strict exclusion criterion for participation, in order to obtain a sample that reflected the disparity of characteristics of families in the general population. The stipulations for participation were that the child was aged between 2 and 16 years and had had received a clinical diagnosis of ASD through Children and Adolescent Mental Health Services (CAMHS), ASD diagnosis was confirmed verbally by the parents. Parents looking to acquire a family pet dog were recruited on a voluntary basis via Dogs for the Disabled’s PAWS (Parents Autism Workshops and Support) network (Dogs for the Disabled 2013). The PAWS program involves a series of three professional workshops that educate parents about dog behaviour, welfare, and training, whist advising on the suitability of, and integration of pet dogs into families with children with ASD. In addition, postings on websites and social networks related to Dogs for the Disabled and the National Autistic Society (NAS), and word of mouth were used to increase the number of participants. Demographic data relating to the child, dog and family were collected. A control group of parents who did not acquire dogs were recruited through PAWS and local networks and sampled at matched timescales. All parents confirmed they were the primary carer of the child. All parents in the intervention group acquired a dog during the study (from Baseline to Post-Intervention), all parents in the control group did not acquire a dog, or live with a dog during any of the sampling points within the study.

#### Intervention Group Participants (Families with a Child with ASD Acquiring a Pet Dog)

Ninety-three carers were initially recruited into the intervention group; of these, 82 completed the baseline sample data. Eight of the 11 that dropped out before this time reported that they had decided not to get a dog within the timescale of the study (five of these transferred to the control group), two acquired a dog prior to baseline and so were excluded; the remaining carer was un-contactable.

Sixty carers provided data in both the baseline (BL) and post-intervention (PI) samples. Of the 22 that dropped out between BL and PI, 11 reported that they would not be acquiring a dog within the study timescale (one of these transferred to the control group), two were outside of the PI sampling window, two requested to drop out of the research, four were un-contactable, one got a trained assistance dog and two acquired dogs but subsequently re-homed them prior to the PI sample (reasons: carer #038 reported that the dog was biting the children, carer #079 was allergic to the dog).

Forty-two carers provided data at all three sample points (baseline, post-intervention and follow-up); 18 dropped out between PI and follow up (FU). Of these eight re-homed the dogs (six due to child-dog issues, one due to child problems unrelated to the dog, one due to dog training problems unrelated to the child), one requested to drop out of the research, one was not contactable, and seven were outside of the study timescale (i.e. the date of dog acquisition meant that it was too late to include them in the follow up sample).

In line with the instructions in the PSI manual (Abidin 1995), four carers were removed from the data set as they had extremely low ‘Defensive Responding’ scores, indicating that their responses may be strongly biased to present a favourable impression. Although in a clinical setting this score would be assessed in relation to other information obtained about the individual, in this study additional information was very limited and so these subjects were removed as a precaution. This left a sample size of n = 38 for repeated measures analysis.

Three carers had a single missing data point, due to an item not being completed. Missing data were calculated as per instructions in the PSI manual (Abidin 1995), by computing the average score for the completed items in the subscale and rounding the average to the nearest whole number.

#### Control Group Participants (Families with a Child with ASD Not Acquiring a Pet Dog)

Thirty-two carers were recruited for the control group, of these 28 completed all three samples. Three of the four who dropped out had acquired a dog (one before baseline who transferred to the intervention group; one before post-intervention; and one before follow-up). The fourth completed two interviews but was un-contactable for the follow up.

Four carers were removed from the data set as a precaution due to extremely low ‘Defensive Responding’ scores, as per the procedure used with the intervention group. From the intervention group 94.7 % (n = 36) were recruited via the PAWS network and 5.3 % (n = 2) from other adverts. Among the control group 37.5 % (n = 9) were recruited from the PAWS network and 62.5 % (n = 15) through contacts with local autism networks.

### Demographics

Demographic characteristics of the intervention (n = 38) and control group (n = 24) are reported in Table 1. There was no significant difference in carer gender between groups (*p* = .640, two-tailed Fisher’s exact test) and no significant difference in number of carers between groups (χ^2^ = 0.231, df = 1, p = .631). There was no significant difference in subject gender between groups (*p* = .752, two-tailed Fisher’s exact test). Participants ages ranged from 2–16 years (Intervention Group: 8.76 years ± 2.86; Control Group: 9.25 years ± 4.06; Mean ± SD). There was no significant difference in participant age between groups (*t*(60) = 0.553, *p* = .582). There was no significant difference in number of siblings between groups (Likelihood ratio = 7.416, df = 4, *p* = .115). All participants had a confirmed diagnosis within the ASD spectrum. There was a significant difference in diagnostic types between groups (Likelihood ratio = 8.034, df = 3, *p* = 0.045) with a greater proportion of Asperger’s/High functioning autism in the dog-acquiring group compared to controls. Language ability was reported by parents on a Likert scale designed by the autism professionals advising on this study. The scale asked parents to define their child’s language skills from: 0 (no language), 1 (single words and gestures), 2 (simple sentences and phrases) 3 (full sentences). The reported language ability varied between participants, however, the reported language ability of the participants was not significantly different between groups (Likelihood ratio = 2.354, df = 3, *p* = 0.502). There was no significant difference in the sampling times between the groups (Table 2).

**Table 1:** Demographic characteristics of the intervention and control group

Demographic item	Intervention (total n = 38)	Control (total n = 24)
Female children	8	4
Male children	30	20
Female parents	34	23
Male parents	4	1
One parent household	9	7
Two parent household	29	17
No siblings	5	8
One sibling	22	8
Two siblings	7	7
Three siblings	2	0
Four siblings	2	1
Autism diagnosis	20	20
Asperger’s/high functioning autism	17	3
Other	1	1
No language ability	1	3
Single words/gestures	5	4
Simple phrases/sentences	8	4
Full sentences	21	13
Language not reported	3	0

**Table 2:** Interval times for sample populations

	Intervention (mean ± standard error)	Control (mean ± standard error)	Independent samples *t* test
BL-PI (days)	Range 28–144 (76.13 ± 34.44)	Range 54–171 (76.96 ± 23.93)	*t* = .158 *p* = .875
PI-FU (days)	Range 144–245 (188.55 ± 21.56)	Range 98–208 (186.21 ± 23.20)	*t* = .405 *p* = .687
BL-FU (days)	Range 177–344 (264.66 ± 35.75)	Range 248–277 (261.08 ± 8.52)	*t* = .590 *p* = .558

*BL* baseline, *PI* post-intervention, *FU* follow-up

Within the intervention group, 36 families acquired a single dog and two families acquired two dogs at the intervention time. The dogs’ age at acquisition ranged from 1.75 to 84 months (5.09 ± 13.62 months; Mean ± SD). There were 17 male dogs and 23 females of these 15 were crossbreeds and 25 were purebred from 11 different breeds: 8 Labrador Retrievers (two acquired by one family), 3 Golden Retrievers, 3 German Shepherd Dogs (two acquired by one family), 2 Cavalier King Charles Spaniels, 2 Miniature Schnauzers, 2 Cocker Spaniels, 1 Sussex Spaniel, 1 Jack Russell Terrier, 1 West Highland White Terrier, 1 Border Collie and 1 Bernese Mountain Dog. 85 % (n = 34) were acquired from breeders; 10 % (n = 4) from rescue; 5 % (n = 2) from other sources.

### Data Collection

To measure parental stress we administered the Parenting Stress Index (III edition), Short Form (Abidin 1995); an abbreviated version of the original 120 item form it is comprised of 36 questions, which takes approximately 10 min to complete. The 36 questions measure three domain of stress; 12 items measure Parental Distress (PD) (e.g. “I often have the feeling that I cannot handle things very well”), 12 items Parent–Child Dysfunctional Interaction (P-CDI) e.g. “My child rarely does things for me that make me feel very good”), and 12 items measure Difficult Child (DC) (e.g. “My child seems to cry or fuss more often than other children”), which combine to provide a score of Total Stress (TS). Items are scored on a five point scale from ‘Strongly Agree’ to ‘Strongly Disagree’. Seven items from the PD scale are summed together to give a score of defensive responding. The PSI short form is suitable for use with the parents of children aged from 1 month to 12 years (the demands and requirements of caring for a child with ASD ensure this scale is suitable for children who may be older than 12 in years, but do not perform as a typically functioning older child would do). The short form was designed for use by clinicians working under time restrictions in primary health care settings and for research purposes. The scales have satisfactory test–retest reliability scores (TS: .84, PD: .85, P-CDI: .68, DC: .78) and good internal reliability coefficients (α) (TS: .91, PD: .87, P-CDI: .80, DC: .85) (Abidin 1995).

The PSI-SF was completed by the primary carer via telephone interview with the researcher at three sample points: Baseline (up to 17 weeks before acquiring a dog in the intervention group); post-intervention (3–10 weeks post dog acquisition); and Follow-up (25–40 weeks post dog-acquisition). Control group participants were sampled at matched timescales (delayed by 8 months to allow for matching by inter-sample duration). A range of demographic data relating to the focal child, family and dog (intervention group) were also collected. A wider range of data was collected as part of this study but only those related to the PSI are reported in this paper.

### Data Analysis

All data was analysed using SPSS software (IBM 2013). Categorical demographic data were presented as frequencies and differences between groups were analysed using Chi squared tests. Continuous demographic data and duration between samples were described by range and mean ± standard deviation and differences between groups were analysed using independent samples *t* tests.

PSI data were checked for normality and found to be normally distributed (Kolgomorov–Smirnov *p* > .05). Summary statistics were calculated and plotted to facilitate visual inspection of results, prior to statistical analysis. Parenting Stress Index scores were analysed using ANCOVAs for Total Stress and each of the subscales: Parental Distress (PD), Parent–Child Dysfunctional Interaction (PCDI) and Difficult Child (DC), with group (intervention versus control) and sample period (post-intervention and follow-up) included as factors, and baseline scores included as a co-variate. Effects of sample time, interaction between group and sample time, and between group effects were reported as significant at *p* < .05. Post-hoc paired *t* tests used to determine the location of significant differences between sample points.

As the primary aims were to assess differences in scores between groups and the clinical relevance of changes in scores, the normality of scores was investigated further, where there was evidence to suggest that the intervention reduced mean scores to within clinically normal values. We defined a clinically high and normal range based on the values provided in the PSI manual (Abidin 1995). Changes within groups in the proportion of carers scoring within a normal range at baseline versus follow up were assessed using McNemar’s Chi squared tests.

In order to assess the impact of the intervention in relation to baseline stress levels, the relationship between baseline Parental Distress, and reduction of Parental Distress from baseline to follow-up, was assessed within groups using Pearson’s correlation coefficients.

## Results

We present the results of statistical analysis on parents’ responses to the items on the PSI-SF separately for each sub-scale. Each section starts with the results of the ANCOVA, followed by consideration of whether the two groups had made a clinically significantly change in their responses over the sampling points.

### Parenting Stress Index Scores

#### Total Stress

**Fig. 1:**
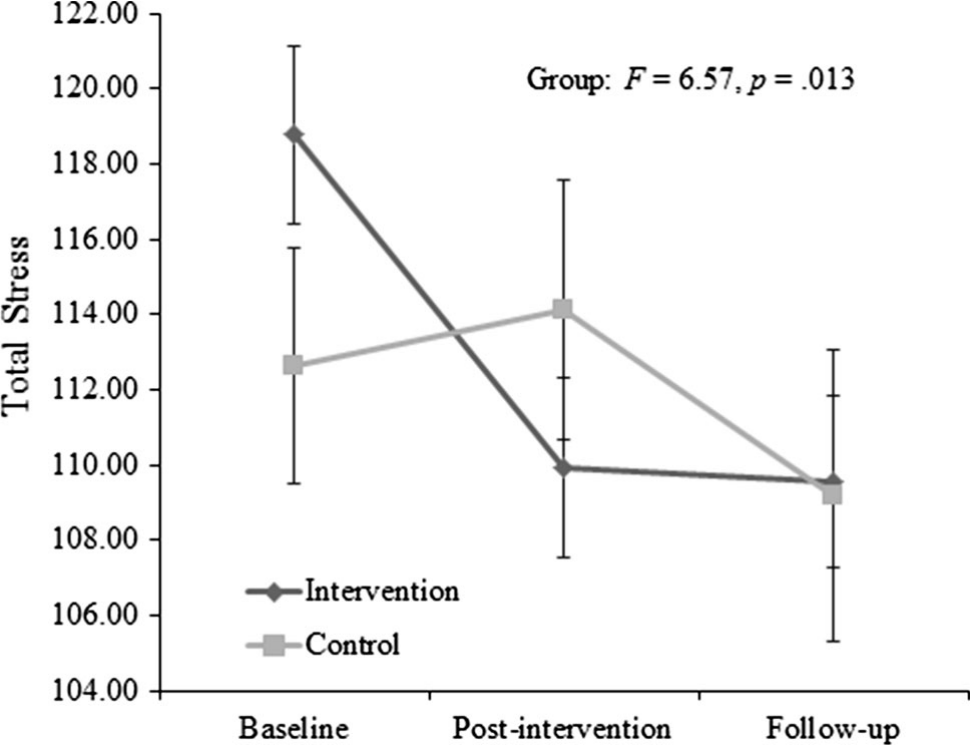
Parenting Stress Index (short form): Total Stress score for intervention group (carers acquiring a dog) and control group (carers not acquiring a dog). Error bars represent ±1SE. Scores are considered clinically high above the 85th percentile (score >86)

There was no significant effect of time on Total Stress scores *F*(1, 59) = .051, *p* = .822. There was also no significant interaction between group and time *F*(1, 59) = 2.448, *p* = 0 = .123. However, there was a significant difference between groups when accounting for baseline scores *F* = 6.568, *p* = .013, η2 = .100. The plot of scores suggested a numerically greater decrease in the intervention group (Fig. 1). Post-hoc pairwise comparisons within groups revealed significant changes in the intervention group between baseline and post-intervention *t*(37) = 4.597, *p* = .000, and between baseline and follow up *t*(37) = 4.048, *p* = .001) but not between post-intervention and follow-up *t*(37) = .200, *p* = .843. Within the control group there were no significant changes between time points (adjusted significance threshold *p* = .017): baseline to post-intervention *t*(23) = −.723, *p* = .477), post-intervention to follow-up *t*(23) = −2.205, *p* = .038) and baseline and follow-up *t*(23) = −2.361, *p* = .027).

Both groups remained well within a clinically high range (>85th percentile/>score of 86).

#### Parental Distress

**Fig. 2:**
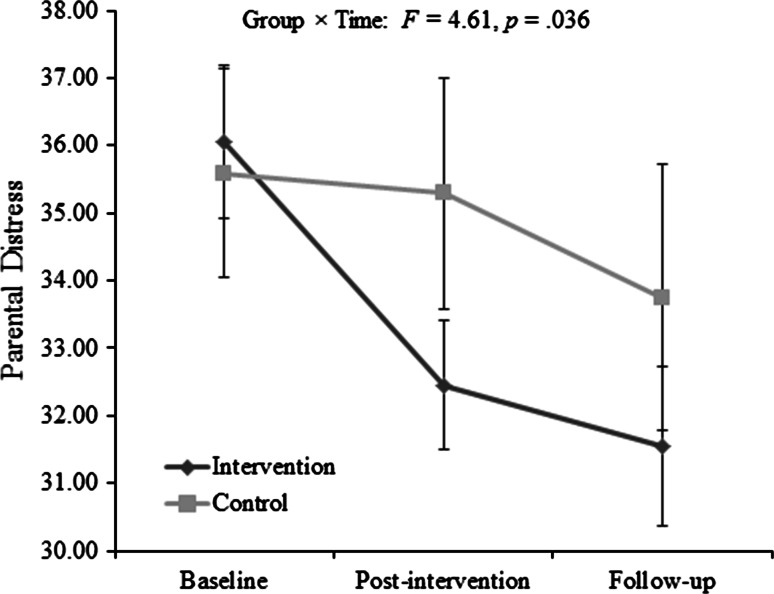
Parenting Stress Index (short form): Parental Distress subscale score for intervention group (carers acquiring a dog) and control group (carers not acquiring a dog). Error bars represent ±1SE. Scores are considered clinically high above the 85th percentile (score >33)

There was no significant effect of time on Parental Distress subscale scores *F*(1, 59) = .439, *p* = .510. There was also no significant interaction between group and time *F*(1, 59) = .175, *p* = .677. However, there was a significant difference between groups when accounting for baseline scores *F* = 4.617, *p* = .036, η2 = .073. The data plot suggested a greater numerical decrease in the intervention group (Fig. 2). The decrease in the intervention group was significant between baseline and post-intervention *t*(37) = 3.988, *p* < .000 and between baseline and follow up *t*(37) = 3.657, *p* = .001, but not between post-intervention and follow-up *t*(37) = 0.994, *p* = .327). There were no significant changes between time points within the control group.

A significant number of carers moved from a clinically high to clinically normal range (at or below the 85th percentile) within the intervention group (McNemar’s *p* < .001) but not within the control group (*p* = .125).

A significant correlation between baseline PD and the reduction in PD was identified for the intervention group (*r* = .497, *p* = .002) but not for the control group (*r* = −.067, *p* = .757).

#### Difficult Child

**Fig. 3:**
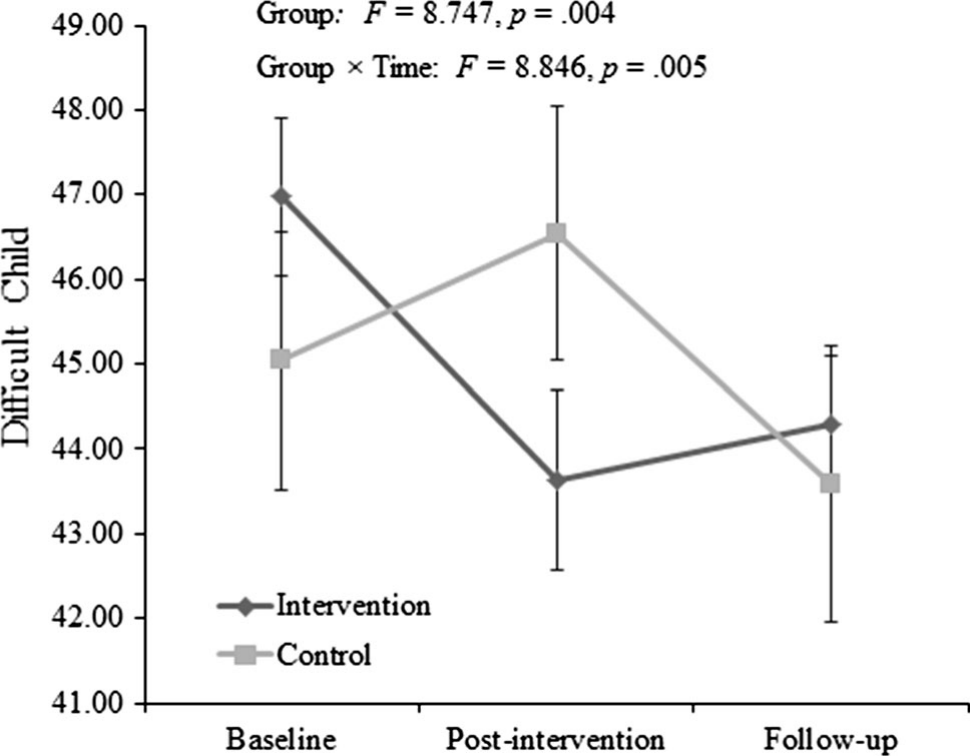
Parenting Stress Index (short form): Difficult Child subscale score for intervention group (families acquiring a dog) and control group (families not acquiring a dog). Error bars represent ±1SE. Scores are considered clinically high above the 85th percentile (score >33)

There was no significant effect of time on the Difficult Child subscale *F*(1, 59) = .173, *p* = .679. There was a significant interaction effect between group and time *F*(1, 59) = 8.846, *p* = .005; Fig. 3). There was also a significant difference between groups when accounting for baseline scores *F* = 8.747, *p* = .004, η2 = .129. The data plot suggests the groups were affected in different ways (Fig. 3). In the intervention group, a significant reduction was apparent between baseline and post-intervention *t*(37) = 4.429, *p* < .000, and between baseline and follow up t(37) = 3.540, *p* = .001) but not between post-intervention and follow-up *t*(37) = −0.749, *p* = .459). Whereas for the control group, there were no significant changes from baseline to post-intervention, *t*(23) = −1.736, *p* = .096), but a significant decrease from post-intervention to follow up *t*(23) = −3.832, *p* = .001); over the whole time course of the study, there was no significant decrease (i.e. from baseline to follow up) *t*(23) = −2.159, *p* = .042). Both groups remained within the clinically high range (>85th percentile/ >Difficult score of 33).

#### Parent–Child Dysfunctional Interaction

**Fig. 4:**
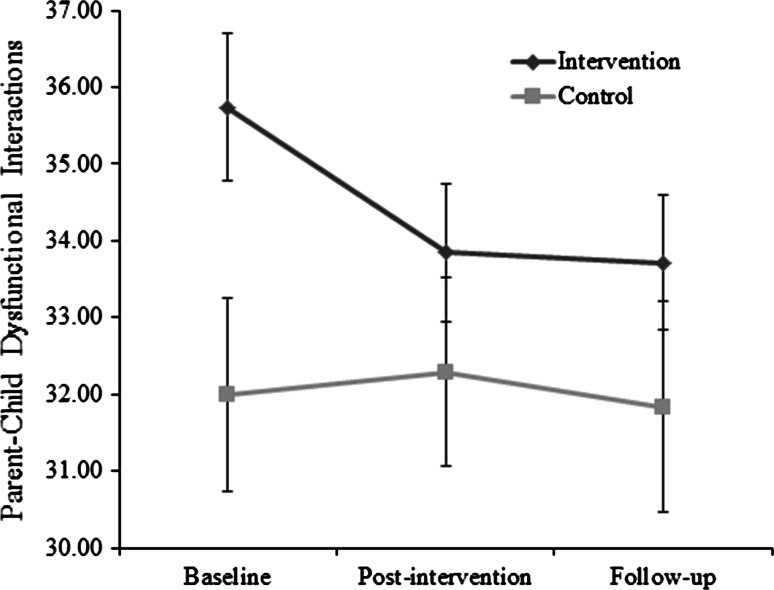
Parenting Stress Index (short form): Parent-Child Dysfunctional Interaction subscale score for intervention group (families acquiring a dog) and control group (families not acquiring a dog). Error bars represent ±1SE. Scores are considered clinically high above the 85th percentile (score >26)

There was no significant effect of time on the Parent–Child Dysfunctional Interaction subscale *F*(1, 59) = .088, *p* = .768 and no significant interaction effect between group and time *F*(1, 59) = .039, *p* = .845. There was also no significant difference between groups *F*(1, 59) = .609, *p* = .438, (Fig. 4). Both groups remained well within a clinically high range (>85th percentile/ >score 26).

## Discussion

A significant effect of group was seen in three out of four of the measures of stress in the Parenting Stress Index. In the intervention group (those acquiring a pet dog) a reduction of parental stress in the domains of Total Stress (overall stress in the parenting system), Difficult Child (the basic behavioural characteristics of the child) and Parental Distress (the level of stress experienced by the carer in their parenting role), these effects were not observed in the control group. Unsurprisingly given the nature of the intervention, the stress associated with the parent’s perception that his or her child does not meet their expectations did not significantly change in either group (Parent–Child Dysfunctional Interaction subscale).

For all three of the measures in which there were significant reductions in the intervention group, these were evident from the post-intervention period and maintained at the lower level at follow-up, suggesting that dogs may provide a relatively immediate stress buffering effect and that this benefit is relatively enduring. For one measure within the intervention group (Parental Distress) values even fell to within clinical normal levels, but such a dramatic change was not seen in relation to the other measures, which remained well within clinically high ranges. The correlation between the magnitude of the reduction recorded in parental distress scores and baseline parental distress scores is consistent with change within the intervention group being specific and not random, and is supported by no such correlation in the control group. This suggests that pet dogs may be more effective at reducing the stress of carers experiencing higher levels of distress associated with the parenting role. In contrast, the only significant changes apparent within the control group (Difficult Child subscale) appear to have been the result of random change, potentially associated with regression to the mean, following a numerical rise post-intervention.

This study is highly original in at least two ways. Previous studies have examined the effect of interventions on parents (Robbins et al. 1991; Remington et al. 2007; Shields and Simpson 2004; Tonge et al. 2006), but none have focused on the introduction of a family dog on parental functioning. Those studies that have examined the impact of dogs on families such as those used in this study, have focused on the effects on the child (Grandgeorge et al. 2012; Carlisle 2014) rather than their incidental effects of pets on carers of the wider family.

These results suggest that stress in primary carers arising from the behaviour of the child and the carer’s global assessment of their situation but not the severity of their assessment of their child against their expectations, are reduced in the medium term as a result of pet dog acquisition. This impact on carer distress may come about via one or more mechanisms, which may vary between individuals. Firstly, there is evidence to support general but direct physiological stress reducing effects arising from interaction with pets (Odendaal and Meintjes 2003; Nagasawa et al. 2009; Katcher et al. 1983). Dogs may also provide social support, which has been suggested to be particularly effective in reducing stress in carers of children with ASD (Koegel et al. 1992; Konstantareas and Homatidis 1989; Weiss et al. 2013). Therefore, carer stress might also be reduced as a result of the social support provided by pet dogs.

Other possible mechanisms for stress reduction may come about more indirectly for example via changes in physical activity, outdoor access, change in routines or carer time away from child, all of which carers are likely to experience following dog acquisition. Indeed, the acquisition of a pet dog is likely to alter family dynamics such as these, which could reduce parental stress. It could be argued that similar changes in dynamics, and therefore parental stress, could be observed in the addition of another human family member, such as a new baby. However, we do not consider this to be a reliable reflection of the mechanisms underlying the improvements in stress levels. For instance, a study by Allen et al. (1991) showed that stress levels reduced greater in the presence of a dog compared to a friend; indicating the unique processes involved in human-animal interactions as opposed to human interactions. Furthermore, animals are unique in the non-judgemental, unconditional support with which they provide (Kruger et al. 2004). Even less direct mechanisms for stress reduction on the primary carer, include the consequences of the positive effects of the dog on other family members and the child with ASD, which have been reported in other studies (Berry et al. 2013). Accordingly these results pose the intriguing possibility that at least some of the previously reported benefits to the child and family of interventions may be largely subjective arising from reductions in parental stress, rather than real changes in child behaviour.

It is likely that different factors are important in different contexts and while this may pose challenges for assessing or inferring the mechanism behind the changes noted; from a clinical perspective this may indicate that dogs are particularly useful, since their nature means they may provide a personalised and socially valid intervention with the minimum of clinical effort. In this context, voluntary dog acquisition offers a flexible intervention that is economically effective, integrates well into the family, making them potentially very useful for the varied symptoms of ASD, which is highly individual in nature.

One limitation with the study is that it is difficult to make direct comparisons with other studies measuring parental stress levels in ASD programs. Previous studies have reported reductions on PSI outcome measures through implementation of parent-focused early intervention programs (Robbins et al. 1991; Shields and Simpson 2004), direct comparisons with these interventions are difficult since there are a number of demographic and methodological differences. These include child age range, baseline score, version of the PSI used and lack of suitable control groups. An important finding to highlight from this study is that pet dogs appear to have greater stress buffering properties for parents who are experiencing higher levels of parental distress to begin with. This is crucial because it directly targets previously identified issues which show that parents with high stress levels do not respond as well to intervention programs (Robbins et al. 1991; Osborne et al. 2008). In contrast, it appears that parents with high stress levels benefit the most from pet-dog intervention programs. Additionally, the introduction of a dog to the family may not only be beneficial in its own right, but also facilitate increased efficacy of other interventions. These results also imply that pet (and especially dog) acquisition needs to be recorded and its impact either controlled for or considered in other longitudinal studies.

A second limitation of this study concerns the prospective case–control design. Pet dogs are widely considered to be family members by their owners, and so while acquisition of a dog represents a voluntary change, or intervention within the family, the nature of this means a randomised intervention design is neither ethical nor feasible. A prospective case–control design is therefore the most rigorous practical design for assessing the effect of this type of change in lifestyle, with the results strengthened by the use of a previously validated clinical scale (Abidin 1995). In order to gain a wide ranging sample to reflect the large individual differences in the diagnosis of ASD the groups were not controlled for diagnosis or child behaviour. It is noted that a greater proportion of the intervention group were recruited though the PAWS network compared to the control group and although there were more children with a diagnosis of HFA/Asperger syndrome in the intervention group, there is currently no consensus on the relationship between parental stress and severity or presentation of ASD (Pisula 2011), and this did not result in significant differences in other demographics or baseline PSI measures between groups, indicating that the groups were generally well matched. Although a potentially important limitation could relate to the loss of data from carers who relinquished a dog after acquisition given the repeated measures design, this concern can be mitigated by considering that the results relate only to families in which there is a successful and voluntary dog adoption. At 16 % within the first 6–8 months, the rate of relinquishment in this study does not appear to be excessive when compared to typical populations of families acquiring dogs (Mondelli et al. 2004; Patronek et al. 1995; Wells and Hepper 2004).

Other limitations of this study include the measures implemented to confirm child diagnosis and language ability. The diagnosis of ASD is subjective and controversial (e.g. Shattuck and Grosse 2007; Szatmari 2011), however, we chose to take parents’ verbal confirmation that their child had received a clinical diagnosis of ASD, and their subjective rating of their child’s language ability as satisfactory evidence for the purpose of this study. This approach leaves open the possibility of misjudgement, for both under and over representation of their child’s functioning. Future studies could explore the significance of using standardised clinical measures of these factors in relation to the effects of pet dogs in these families to assess whether the strength of the findings is increased or decreased.

While these findings are of enormous potential impact, it has to be recognised that they relate only to the first 6–8 months post dog acquisition and there is a need for longer term follow up. This is a priority, given how promising the results appear, since it is necessary to establish both whether the reduction is maintained and/or continues to help normalise the stress within families in the longer term. It should also be noted that, in this study, the majority of families acquired dogs as young puppies which may also be relevant to the impact seen. However, it would be expected that puppies would place more demands on the carers, in terms of the amount of management and training required, and so while they may be more beneficial in some ways, they may also be more demanding. Despite this, the results of this study indicate that clinicians should support families thinking about getting a dog, so long as realistic expectations are set via programs like PAWS and no specific additional risks are identified.

These results also provide a solid basis to raise other important questions in relation to the potential for dogs to provide stress-ameliorating effects to the wider carer population, for example those caring for sufferers of dementia, or the terminally ill, which is an area of growing economic and political concern.
